# An Efficient Retrieval System Framework for Fabrics Based on Fine-Grained Similarity

**DOI:** 10.3390/e24091319

**Published:** 2022-09-19

**Authors:** Jun Xiang, Ruru Pan, Weidong Gao

**Affiliations:** School of Textile Science & Engineering, Jiangnan University, No. 1800, Lihu Avenue, Wuxi 214122, China

**Keywords:** fabric retrieval, deep hashing, fine-grained similarity, variational network, similarity embedding

## Abstract

In the context of “double carbon”, as a traditional high energy consumption industry, the textile industry is facing the severe challenges of energy saving and emission reduction. To improve production efficiency in the textile industry, we propose the use of content-based image retrieval technology to shorten the fabric production cycle. However, fabric retrieval has high requirements for results, which makes it difficult for common retrieval methods to be directly applied to fabric retrieval. This paper presents a novel method for fabric image retrieval. Firstly, we define a fine-grained similarity to measure the similarity between two fabric images. Then, a convolutional neural network with a compact structure and cross-domain connections is designed to narrow the gap between fabric images and similarities. To overcome the problems of probabilistic missing and difficult training in classical hashing, we introduce a variational network module and structural module into the hashing model, which is called DVSH. We employ list-wise learning to perform similarity embedding. The experimental results demonstrate the superiority and efficiency of the proposed hashing model, DVSH.

## 1. Introduction

**Figure 1:**
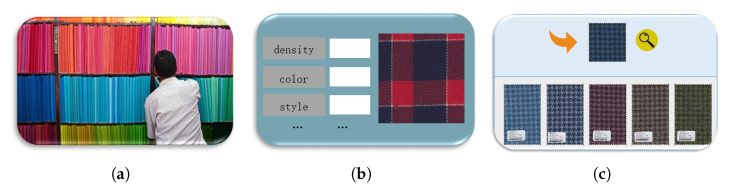
Three kinds of fabric image retrieval methods. (**a**) Real sample search; (**b**) text-based image retrieval; (**c**) search by image.

In the context of the major strategy of “carbon compliance and carbon neutrality”, as one of the traditional industries with high energy consumption, the textile industry is faced with the severe challenge of energy conservation and emission reduction. Improving the intelligence, digitization and automation level of textile enterprises are effective measures to help enterprises save energy and reduce emissions. In addition, with “small batch, multi-variety, tight delivery” increasingly becoming the main production mode of textile enterprises, enterprises have accumulated large amounts of historical production data. How to quickly locate target data in a large amount of data and use them to guide production has become an urgent problem for textile enterprises. At present, there are two methods of fabric retrieval commonly used by textile enterprises: real sample search and text-based image retrieval (TBIR). The former method stores the real samples of fabrics, and the retrieval is carried out by manual comparison, as shown in Figure 1a, which not only takes up storage space, but also has low efficiency and strong subjectivity in manual comparison and retrieval. In addition, textile fabrics will fade with the extension of storage time, which will affect the retrieval results. As shown in Figure 1b TBIR performs fabric retrieval in a semi-manual manner, using human annotations to index fabric images. Although it overcomes the shortcomings of real sample search to a certain extent, the labeling process still makes the retrieval results subjective, resulting in limited retrieval accuracy, and the retrieval methods that can be provided are relatively simple. Content-based image retrieval (CBIR) uses a specific visual representation method to index image content, as shown in Figure 1c, which can overcome the shortcomings of the above methods, so it has become a research hotspot in this field. In this paper, we propose a novel CBIR method to achieve accurate fabric image retrieval.

When receiving a query image, the CBIR system is expected to output a list with the same visual content as the query. Technically speaking, there are two phases in the CBIR, namely, image representation and feature matching. Image representation vectorizes the input images (including queries and images in the database), and the second phase ranks the images in the database for similarity and outputs similar images. Currently, the most challenging task in CBIR is to associate pixel-based, low-level features with human-perceived, high-level semantic features. In many previous works [1,2,3], some hand-crafted feature descriptors were used to represent the visual content of fabric images, such as SIFT [4], LBP [5] and Color Moment [6]. Even though these pixel-level methods have achieved some success, they rely too much on feature engineering, which leads to their limitations in robustness. Recently, convolutional neural networks (CNNs) have achieved outstanding performance in many vision tasks, such as image classification, person identification [7,8], image segmentation and object detection, which demonstrate its good performance in visual description. Therefore, many researchers adopted CNNs for image retrieval tasks. CBIR has seen a significant breakthrough due to the replacement of earlier low-level feature-based algorithms with an end-to-end framework based on deep learning. Inspired by this trend, we focus on the use of deep CNNs to solve the problem of fabric image retrieval. Krizhevsky et al. [9] directly used the convolutional layer in a CNN as the index for the image, and its excellent retrieval performance demonstrated the superiority of deep CNN for image retrieval. However, the disadvantage of this method is its high computational cost, which resulted in a long retrieval time.

To improve retrieval efficiency, a lot of feature optimization and encoding methods were proposed, of which the most commonly used approach was the approximate nearest neighbor search (ANN). At present, deep hashing [10,11,12], which is designed to automatically learn the optimal hash function and generate image hash codes, has become the most popular ANN method. The deep-hashing-based method searches nearest neighbors by measuring the similarity in Hamming space between the generated hash codes.

Differently from general natural images, the abstraction of semantic features in fabric images is not high, which mainly include color, texture and higher-order features formed by their interaction. The different feature types in images make it difficult for general image retrieval methods to directly apply fabric image retrieval. To achieve efficient fabric image retrieval, many researches represented the visuals of fabric images by using hand-crafted feature descriptors, and achieved good performance. However, the success of hand-crafted methods is limited to small datasets or specific fabric types. Our previous work [13,14] attempted to use classification tasks to guide CNN models to learn fabric image representations. Although the retrieval performance far exceeded those of other low-level feature-based methods, the retrieval accuracy still fell short of the retrieval requirements of textile enterprises. There are two main reasons for this problem: (1) the similarity of fabrics cannot be measured by rough classification; (2) the feature loss is severe in the hashing process. To address the problem, in this paper, we first design a fine-grained similarity to measure the similarity between fabrics, and then introduce the structural network and the variational network in the hashing process to reduce the feature loss. Specifically, to narrow the gap between fabric images and similarities, we design a CNN with a compact structure and cross-shortcut connections, which is regarded as the base network of the hashing model. To overcome the problems of probabilistic missing and difficult training in classical hashing, we introduce a variational network module and structural network module into the hashing model (named DVSH). Then, a fine-grained similarity is defined to measure the similarity between two fabric images. To incorporate the defined fine-grained similarity into hash learning, we employ list-wise learning to complete similarity embeddings in mini-batches.

## 2. Motivation

The goal of image retrieval is to quickly and accurately retrieve relevant images from the target database. In this paper, we believe that two key issues in this task are: (1) how to define the similarity between two images; (2) How to efficiently retrieve relevant images.

At present, there are generally two ways to define similarity between images, namely, binary pairwise similarity [15,16] and soft similarity [17,18]. The former has two ways of measuring similarity: (1) two images are considered similar if they share at least one label—otherwise, they are dissimilar; (2) if the labels of the two images are completely identical, they are considered similar—otherwise, dissimilar. Due to the loss of too much information, the two definitions of binary pairwise similarity are not suitable for measuring the similarity between fabric images. Regarding soft similarity, it is calculated by the degree of fit or cosine distance between the label matrices, which needs to be established on the basis that the categories are independent of each other. For fabric images with multi-granularity features, which contain features at multiple levels, the above similarity definition methods are not applicable. This paper argues that an ideal measure of fabric similarity should be designed according to some well-designed rules, and the similarity of each dimension should be considered. Therefore, according to the characteristics of fabric images, we designed a fine-grained similarity.

**Figure 2:**
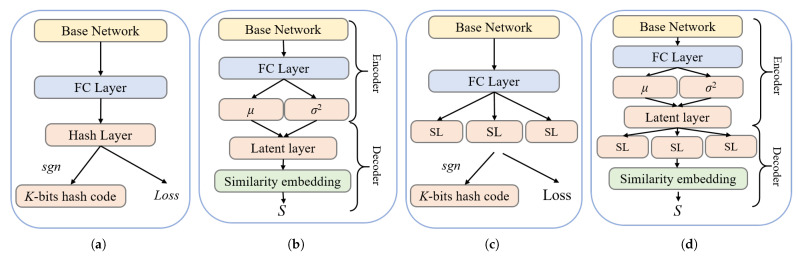
Four different hash network architectures. (**a**) Typical hashing network; (**b**) variational network; (**c**) structural network; (**d**) variational and structural network.

Generally, the dimensionality of the features extracted by a CNN is high, which leads to a large computational cost in feature matching (called the “curse of dimensionality”). To solve this situation, some researchers proposed to use principal component analysis (PCA) [19] for linear dimensionality reduction of high dimensional features. PCA expects that the variance between the information in the projected dimension is the largest, so as to obtain the largest amount of information in fewer data dimensions. The interpretation of the principal components in the features extracted by PCA has a certain ambiguity, and the components with less contributions may be more effective for the representation of the samples, and whether the orthogonal vector space of the eigenvalues is unique remains to be discussed. Approximate nearest neighbor search (ANN) is currently a more efficient method that has made substantial progress in the past decade, especially in visual search applications. Hashing methods are typical in ANN; however, naive hashing methods are difficult to preserve the similarity of input features due to the limitation of code length. With the development of deep learning, deep hashing (DH) methods have achieved rapid development. Deep hashing methods map real vectors in Euclidean space to Hamming space. Efficient searching can be achieved by using the generated binary hash codes, which preserves similarity information. The current commonly used deep hashing network consists of a base network and a hash layer, which is guided by a specific objective function, as shown in Figure 2a. The adopted base network generally consists of some basic types of layers (e.g., convolutional and pooling layer), which are used for abstraction and optimization of features. The hash layer generates binary hash codes. This architecture has two drawbacks: first, although it can learn nonlinear features, such models are not based on probabilistic modeling, which may limit their ability to learn diverse features; second, the hash layer actually is a bottleneck layer, which is difficult to train using the backpropagation algorithm and has serious information loss.

The deep variational generative model [20] integrates data distribution priors into the deep model by combining deep neural networks and probability theory, and its effectiveness has been proven in tasks such as image recognition and image segmentation. Therefore, we believe that variational networks have the potential to improve the performances of deep hashing models. As shown in Figure 2b, the variational network can be viewed as consisting of an encoder and a decoder. The role of the encoder is to sample the output of the fully connected layer. The stochastic layer makes the latent output come from a variational distribution parameterized by a probabilistic model (defined by μ and $\sigma^{2}$), which provides the model with strong generalizability. The decoder maps the output of the latent layer into the similarity matrix *S*. To learn more information during the training process, this paper introduces the structure layer [21] in the hash model, as shown in Figure 2c. The structure layer contains multiple modules, where each module represents multiple binary hash codes, and the structure layer has higher dimensions (the number of nodes) than the hash layer, so more information can be obtained during training. In the testing phase, the output of each node does not correspond to a one-bit hash code, but the outputs of the modules are quantized and spliced together to obtain the final hash code. Figure 2d shows the hash model that combines the two key technologies.

## 3. Fine-Grained Similarity of Fabric Images

The goal of fabric image retrieval is to search for the most similar image set to the query image from the fabric image database, and the key is how to judge whether two fabric images are similar. In this study, we propose to describe the similarity between fabric images in four different dimensions, namely, coarse-texture, fine-texture, color and tightness. Both coarse-texture and fine-texture are texture features (only the observation scale is different). Color is represented by the composition and distribution of colors in a fabric image. The tightness is indicated by the tightness index of the fabric. The proposed similarity is measured from these four dimensions. This section describes how the fine-grained similarity is calculated.

### 3.1. Similarity of Textures

**Figure 3:**
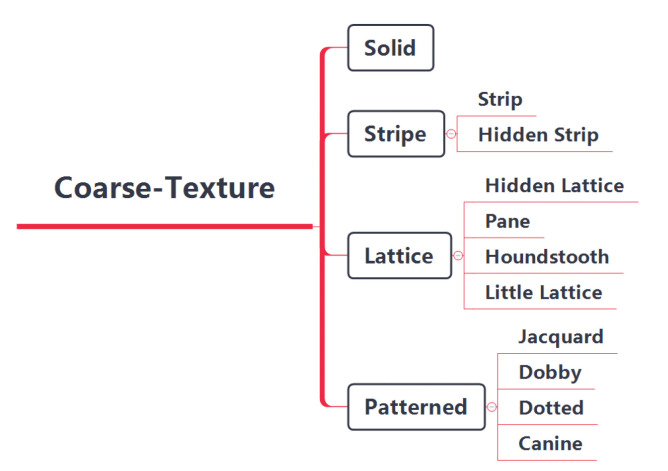
Details about the classification of fabric coarse texture in this study.

**Figure 4:**
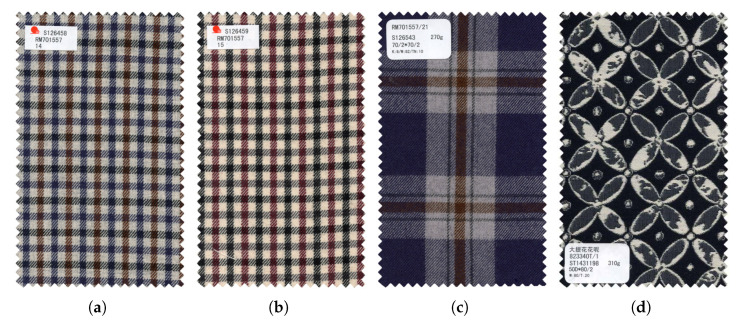
Four examples of fabric images. (**a**,**b**) Small lattice; (**c**) window pane; (**d**) Jacquard (patterns in fabric images are more complex).

In our previous works [13,14], we classified fabric images from coarse-texture and fine-texture perspectives, respectively. Here, we classify coarse textures more delicately, as shown in Figure 3. The coarse texture of the fabric is divided into four major categories: solid color, stripe, plaid and pattern, and each category is subdivided into different numbers of minor categories. Visually, the similarity between fabrics with the same major category but different minor categories is lower than the similarity between fabrics with the same minor category and higher than the similarity between fabrics with different major categories. Therefore, we define three levels for the similarity of coarse textures, namely: similar, approximate and dissimilar. Quantitatively, the similarity between similar fabric images is defined as 1, the similarity between approximate fabric images is 0.5 and the similarity between dissimilar fabrics is 0. The four fabric image samples are shown in Figure 4, in which both (a) and (b) are small lattices, and their similarity in coarse-texture is 1; (a) and (c) are lattice fabrics, but (c) is a pane with different grid sizes, and their similarity in coarse-texture is 0.5; (a) and (d) belong to different categories, and their similarity is 0. This method avoids the drawbacks of the commonly used non-0 or 1 similarity definition method, and makes the similarity exist in a “middle zone”, which is more in line with human’s cognition of the similarity of things.

Visually, the differences between fine-textures are obvious. Therefore, for the similarity of fine-texture, this paper simply adopts the commonly used binary pairwise similarity matrix; that is, the similarity between fabrics in the same category is 1, and the similarity between fabrics in different categories is 0.

### 3.2. Similarity of Colors

Color is one of the most important characteristics of fabrics. In the image, the color of each pixel is converted into a numerical representation and mapped into the RGB space, and each color can be represented by a three-dimensional vector, so the color is the underlying feature at the pixel level. However, the RGB color model was proposed from a hardware perspective, which has poor uniformity and is difficult to match with the visual features observed by the human eye. Compared with RGB, HSV is closer to the human eye’s perception of color, so this color model is often used for color feature extraction in image processing. The name of HSV consists of the first letters of the three components: H for hue, S for color saturation and V for color brightness. This way of defining colors is more suitable for comparisons between colors. Here, referring to OPENCV, we divide the HSV color space into 10 regions, which are represented by $\left\{c_{1}\cdots c_{i}\cdots c_{10}\right\}$. Except for solid fabrics, other types of fabrics generally contain two or more colors. Before measuring the color similarity of fabric images, the color features need to be quantified first. The quantification process of fabric color is divided into two steps:

(1) First, convert the fabric image in RGB space to HSV space, and then gather the colors of all pixel points into 10 divided areas according to the classification criteria in Table 1;
(2) Then, calculate the value of the cluster center of each clustered area and set the pixel point $x=\{x^{H},x^{S},x^{V}\}$. $c_{i}$ represents the area index where this pixel point is located. Then, the value of the cluster’s center can be represented by the mean of all points in the area class:
  (1) $${\bar{x}}_{i}=\frac{\sum_{x\in c_{i}}x}{\sum_{x\in c_{i}}1}$$

**Figure 5:**
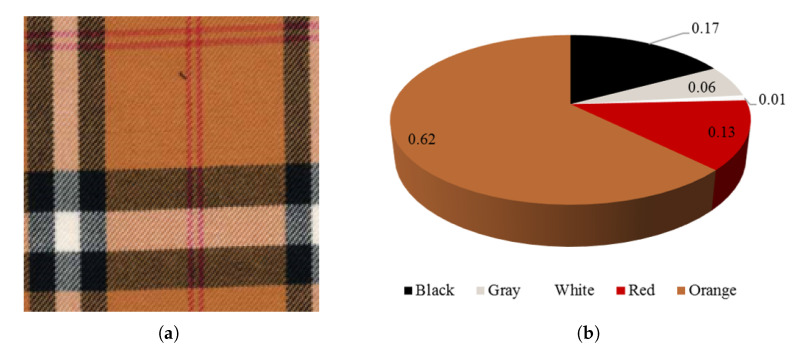
Fabric image color quantification. (**a**) Original fabric image; (**b**) percentages of various colors.

In addition to the value of the cluster’s center, the proportion of each color needs to be calculated. Here, we directly discard the color whose percentage is less than 1%. Then, the color information in the fabric image can be quantified by $C=\{\left(c_{i},{\bar{x}}_{i},p_{i}\right),i=1,2,\cdots N\}$, where *N* is the number of colors. Figure 5 shows an example of fabric image quantization. In the final quantization result, white, which accounts for less than 1%, is discarded. In the final quantization result, the fabric image shown in (a) contains four colors.

For the fabric color information obtained by the above quantization algorithm, it is difficult to use the general color distance calculation method. Assuming that $C_{1}=\left\{\left(c_{i},{\bar{x}}_{i},p_{i}\right),i=1,2\cdots N_{1}\right\}$ and $C_{2}=\left\{\left(c_{j},{\bar{x}}_{j},p_{j}\right),j=1,2\cdots N_{2}\right\}$ represent the color quantization results of two fabric images, respectively, their similarity is calculated by the following equations:

(2) $$S_{C}\left(C_{1},C_{2}\right)=\sum_{i=1}^{N_{1}}\sum_{j=1}^{N_{2}}a_{ij}*S_{ij}$$

(3) $$a_{ij}=\sqrt{\sum_{h,s,v}\left({\bar{x}}_{i}\right)-{\bar{x}}_{j}{)}^{2}}$$

(4) $$S_{ij}=\left[1-\right|p_{i}-p_{j}\left|\right]\times \min\left(p_{i},p_{j}\right)$$

where $a_{ij}$ is the Euclidean distance between the two cluster centers in three color components (HSV). The larger the value of $S_{C}\left(C_{1},C_{2}\right)$, the more similar the two colors are, and vice versa.

### 3.3. Similarity of Tightness

For coarse and fine texture, the categories are discrete, whereas the fabric tightness is actually continuous. It is generally believed that the more similar the tightness between the fabrics, the higher the similarity. Here, we use the original fabric tightness information to measure the similarity of tightness. For two fabrics with tightness values $w_{1}$ and $w_{2}$, their similarity is defined as follows:

(5) $$S_{w}\left(w_{1},w_{2}\right)=1-\frac{|w_{1}-w_{2}|}{\alpha \cdot \eta }$$

The similarity is evaluated according to the difference between the fabric tightness, α represents the distance between adjacent levels and η represents the number of levels. If $w_{1}-w_{2}\geq \alpha \cdot \eta$, it means that there is a big difference between them. In that case, the similarity of the tightness between the two fabrics is regarded as 0. If the difference between $w_{1}$ and $w_{2}$ is small, the value of $S_{w}\left(w_{1},w_{2}\right)$ is close to 1, indicating that they are very similar. In this paper, α=2% and η=20 are configured so that the similarity between fabrics is continuous. This definition of fabric tightness similarity is more refined than the binary pairwise similarity matrix, and is more suitable for describing the similarity between such sequence features.

This section defines the similarity measure of fabrics from four granularities, in which tightness ($S_{w}$) and texture ($S_{ct}$ and $S_{ft}$) are at the semantic level, and color ($S_{c}$) is at the underlying pixel level. Finally, the overall similarity between the two fabric images is expressed as:

(6) $$S=\frac{S_{ct}+S_{ft}+S_{c}+S_{w}}{4}$$

## 4. Deep Variational and Structural Hashing

**Figure 6:**
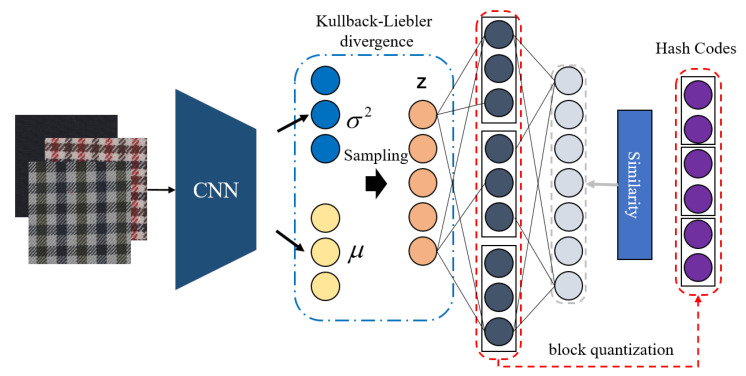
Model structure of deep variational and structural hashing.

Assume $I=\left\{I_{1},\cdots I_{n}\right\}\in R^{d\times n}$ is the training set used to train the hash model, where *I* represents the fabric image, *d* denotes the channel of the images and *n* represents the number of images in the training set. The goal of hash learning is to learn a hash code generator that can convert input image into binary hash codes while embedding similarity information *S* into the generated *K*-dimensional binary hash codes *B*. The hashing process can be expressed as:

(7) $$g:I\mapsto B\in {\left\{-1,+1\right\}}^{K}$$

where *g* denotes the hash function and *K* represents the length of generated hash codes. The framework of the proposed hashing model, which consists of base network module, variational network module and structural network module, is shown in Figure 6 (called DVSH).

### 4.1. Base Network Module

**Figure 7:**
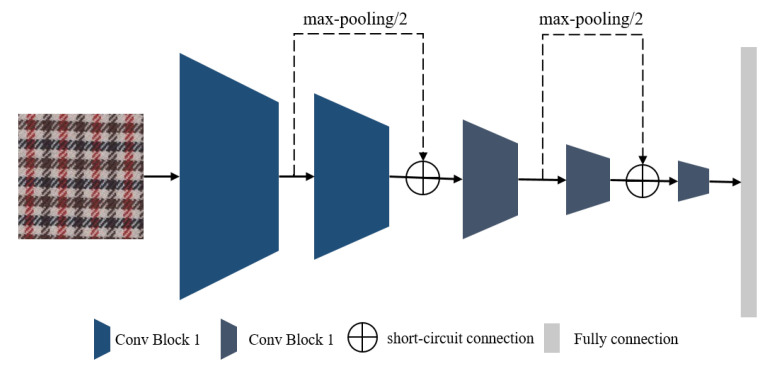
The architecture of the proposed convolution neural network: a compact structure and cross-circuit connections.

Most of the features in the fabric image are some global low-level features, such as color and texture, and the middle and high-level features produced by their combinations. Studies [22,23,24] have shown that the last layer of the deep convolutional neural network contains the highest-order features that can be extracted from this model. The output of this layer is the deep features learned after several convolution operations. The visual content is highly abstract and contains rich semantic information, such as the location, size, and category of the target. These features are the abstraction of the output of the previous layer, so the output of the previous layer has a lower degree of abstraction. Therefore, the features extracted by deeper convolutional layers are more abstract. Generally, the output of the first and second layers often contains rich color and texture features. In our previous study [25], we designed a deep convolutional neural network with a compact structure and cross-circuit connections to bridge the “semantic gap.” The architecture of the proposed CNN is shown in Figure 7, which contains five convolutional blocks, two short-circuit connections and one full connection. Comparative experiments demonstrated the effectiveness and superiority of this CNN for fabric image representation. Therefore, this study directly applied the CNN shown in Figure 7 as the base network, whose details can be found in reference [25]. The parameters in the base network are denoted as $\Theta_{base}$; then the features extracted by the base network can be represented by $r_{i}=\Phi_{base}\left(I_{i},\Theta_{base}\right)$.

### 4.2. Variational Network Module

Inspired by the success of variational encoders, we employed a probabilistic interpretation to the hashing network. The output of base network is regarded as the latent representation $z_{i}$, which is assumed to be represented by its posterior distribution $p_{\theta }\left(z_{i}|r_{i}\right)$. Under this assumption, the posterior probability approximately follows a normal distribution:

(8) $$q\left(z_{i}|r_{i}\right)=N\left(z_{i}|\mu_{i},\sigma_{i}^{2}I\right)$$

By doing a re-parameterization trick, we can sample $z_{i}$ as:

(9) $$z_{i}^{l}=\mu_{i}+\sigma_{n}⊙\epsilon^{l}$$

(10) $$\epsilon^{l}∼N\left(0,1\right)$$

where $\epsilon^{l}$ means the *l*th sample of noise and ⊙ denotes element-wise multiplication; $\mu_{i}$ and $\sigma_{i}$ would be the output of the nonlinear projection from the hashing network. Then, the proposal distribution should follow a prior distribution over the latent variable defined as a multivariate Gaussian:

(11) $$p_{\theta }\left(z\right)=N\left(z;0,I\right)$$

We can enforce this assumption by using Kullback–Liebler divergence (KLD) derived as:

(12) $$L_{KLD}=-KLD(q\left(z_{i}|x_{i}\right)\left|\right|p_{\theta }\left(z\right))=\frac{1}{2}\sum_{j=1}^{J}\left(1+\log\left({\left(\sigma_{i}^{\left(j\right)}\right)}^{2}\right)-{\left(\mu_{i}^{\left(j\right)}\right)}^{2}-{\left(\sigma_{i}^{\left(j\right)}\right)}^{2}\right)$$

where *j* is the *j*th element of μ and σ. The KLD would act as a regularizer of the proposed distribution.

### 4.3. Structural Network Module

From the latent variable, we impose structure in the succeeding fc layer by splitting it into *M* blocks such that $\Phi_{structure}$ is parameterized by $\Theta_{structure}={\left\{\Theta_{struct}^{(m}\right\}}_{m=1}^{M}$. Each block would project the latent sample into a distinct semantic representation. Each block vector is represented as:

(13) $$U_{m,i}=\Phi_{structure}^{\left(m\right)}\left(z_{i},\Theta_{struct}^{\left(m\right)}\right)$$

where $\Phi_{structure}^{\left(m\right)}$ represents the nonlinear projections made on $z_{n}$ for the *m*th block. Assume $U_{m,i}{|}_{m=1}^{M}$ is a struct block with the length of *A*. Then, the shared struct layer output would be:

(14) $$U_{i}=\left[\mathrm{Softmax}\left(U_{1},i\right),\mathrm{Softmax}\left(U_{2},i\right),\cdots ,\mathrm{Softmax}\left(U_{A},i\right),\right]$$

where $U_{i}\in R^{1\times MA}$. Softmax is adopted for each block output to help maximize the potential of one element on each block, which would prevent approximation loss during encoding.

### 4.4. Hashing Learning

In fact, the process of converting from continuous variables to binary hash codes is a process of information loss, which can be regarded as the conversion of continuous variables to Hamming distances through lossy information channels. Channel capacity determines the amount of information that can be transferred from continuous variables. Through theoretical derivation, Li et al. [26] proved that when the input continuous variables obey the bi-half distribution, the channel capacity is the largest and the information loss in the hashing process is the smallest. Following its conclusion, we design the loss function in the hashing process:

(15) $$L_{q}=\sum_{i=1}^{K}\left(\right|u_{i}{\left|-1\right)}^{2}+\left|\sum_{i=1}^{K}u_{i}\right|$$

To incorporate the defined fine-grained similarity into hash learning, we use list-wise learning as the learning method. During the training, the model receives a set of n fabric images, $X=x_{1},x_{2},\cdots ,x_{n}$. $x_{1}$ is regarded as an anchor, and then the similarity matrix $S=\{S_{11},S_{12},\cdots S_{1n}\}$ can be obtained by using Equation (6). Based on the similarity matrix, we can obtain the true permutation of the input fabric images, which is represented by P={P(1),P(2),⋯P(n)}. Then probability of permutation P is expressed by:

(16) $$P_{P}=\Pi_{j=1}^{n}\frac{\phi (d_{P(j)})}{\sum_{k=j}^{5}\phi \left(d_{P(k)}\right)}$$

where $d_{P(j)}$ denotes the distance of image at position *j* of permutation P. Here, we set ϕ(x)=exp(x). During training, the true permutation of the input images is known. Then, according to the idea of maximum likelihood estimation, by maximizing the log-likelihood corresponding to the true permutation (or equivalently minimizing the negative log-likelihood), the parameters of the model then can be optimized, and the loss function can be written as:

(17) $$L_{P}=-\log\left(P_{P}\right)=-\log\left(\Pi_{j=1}^{n}\frac{\phi (d_{P(j)})}{\sum_{k=j}^{5}\phi \left(d_{P(k)}\right)}\right)$$

In summary, the total loss function of the model can be expressed as:

(18) $$L=\lambda_{1}L_{KLD}+\lambda_{2}L_{q}+\lambda_{3}L_{P}$$

where L is the total loss, $L_{P}$ is the list-wise loss of feature *U* and $L_{q}$ is the quantitative loss. $\lambda_{1},\lambda_{2}$ and $\lambda_{3}$ are three weighting parameters to balance the effects of different loss functions. Each sub-item in the objective function is differentiable, so this optimization problem can be regarded as a convex optimization problem. Like other deep learning models, the stochastic gradient descent (SGD) algorithm and the backpropagation (BP) algorithm are used to optimize the parameters in the proposed model.

## 5. Experiments

### 5.1. Experimental Detail

As we all know, the CBIR method based on deep learning learns the image representation ability from a certain amount of training data. Therefore, data are the basis for model learning. In our previous studies [13,14], a dataset named WFID has been established for studying fabric image retrieval. WFID consists of 82,073 fabric images, all of which are annotated from four perspectives: coarse texture, fine texture, fabric style and the pattern forming method. In this study, we still used this dataset to demonstrate the effectiveness and superiority of the proposed method for fabric image retrieval. The difference is that only two versions of annotations were used in this study: coarse texture and fine texture. For fair comparison, the proposed method and compared learning-based methods were all trained on the training set with 33,645 fabric images; the performances of all methods were evaluated on the validation set. The validation set consists of 1029 sets of samples, each of which is an image.

The proposed DVSH was implemented by using the Pytorch toolkit. The hardware environment is as follows: CPU = E5 2623V4@2.60GHz, RAM = DDR4 32G, GPU = GeForce RTX 3090(24G) × 2. All compared deep learning-based methods were implemented using the Pytorch toolkit and based on the backbone of VGG-16. Additionally, the other methods, which are based on hand-crafted descriptors, were implemented by using MATLAB 2018b. During the training, the hyper-parameter configuration was as follows: batch_size = 32, weight_decay = $5\times 10^{-5}$, optimizer = ADAM and learning_rate = $1\times 10^{-3}$.

Generally, CBIR methods are evaluated based on precision, recall or the precision–recall curve. In addition, we also adopted mAP and NDCG to more comprehensively evaluate the performance of each method. We computed each evaluation metric by referring to reference [25].

### 5.2. Parameter Analysis

There are three parameters, $\lambda_{1}$, $\lambda_{2}$ and $\lambda_{3}$, to balance different objective functions. $L_{P}$ is the main driver to guide the learning of the model, so its corresponding weight parameter $\lambda_{3}$ was directly set to 1. $\lambda_{1}$ and $\lambda_{2}$ were, respectively, used to adjust the gradients of the corresponding parameters of the objective functions $L_{KLD}$ and $L_{q}$ during training. In this experiment, the influences of different weight parameter configurations on the final retrieval effect of the model were compared to optimize the optimal parameter configuration. We used the control variable method for analysis and comparison—that is, fixing one of the parameters to analyze and discussing the influence of another parameter on the performance of the model.

The retrieval performance comparison results of the models under different parameter configurations are shown in Table 2.

(1) When the parameter $\lambda_{2}$ is fixed, the performance of the model under each evaluation index shows a similar law; that is, with the increase in $\lambda_{1}$, the performance of the model shows a trend of first increasing and then decreasing. When its value is close to 1, the guiding effect of the corresponding objective function will be stronger than in other tasks; when its value is lower than $10^{-5}$, the guiding effect of the corresponding objective function is too low and is covered by other tasks. Both cases will affect the feature selection ability of the variational network module, which will lead to the degradation of retrieval performance.
(2) When $\lambda_{1}=2$ is fixed, and the value of $\lambda_{2}$ is 1 and $10^{-1}$, each retrieval evaluation index of the model is very low, and the retrieval effect varies greatly in each coding length. This phenomenon shows that the guiding effect of this objective function completely covers other objective functions under this condition, which leads to the instability of the model performance. In particular, the retrieval performance of the model is the worst in Table 2, and it can be considered that the similarity of the fabric images is not embedded in the generated hash code. When the value of $\lambda_{2}$ is in the range of [$10^{-5}$, $10^{-2}$], the retrieval performance of the model is not very different, and all of them can achieve good performance. When $\lambda_{2}<10^{-6}$, the model performance has a certain decline. When the parameters take values in the interval [$10^{-5}$, $10^{-2}$], the sensitivity of the model performance is low, and the performance is relatively stable.

In contrast, the DVSH model is more sensitive to $\lambda_{2}$, because the quantization loss directly affects the quality of the generated hash code. The experimental results show that when $\lambda_{1}=10^{-3}$ and $\lambda_{2}=10^{-3}$, the model achieves the best performance, so this configuration was used in subsequent experiments.

### 5.3. Ablation Study

To gain insight into DVSH, we performed an ablation study. DVSH contains a variational network module, a structural network module and a similarity embedding module. Undoubtedly, the similarity embedding module is an indispensable part of the model, so this module was not analyzed in the ablation study. The other two modules of DVSH are very flexible and can be added or removed. Under the condition that the similarity embedding module is preserved, DVSH produces three variants: DVSH1a (removing the variational network module), DVSH1b (removing the structural network module) and DVSH2a (removing both modules). The comparative experimental results of DVSH and its three variants are shown in Table 3. It is clearly observed that removing any one of the modules in DVSH leads to a decrease in model performance. The performance of DVSH2a with both modules removed is significantly reduced. The experimental results demonstrate that the structural network module and the variational network module can jointly promote hash learning, so that the generated hash codes retain more useful information.

### 5.4. Time Complexity Analysis

**Figure 8:**
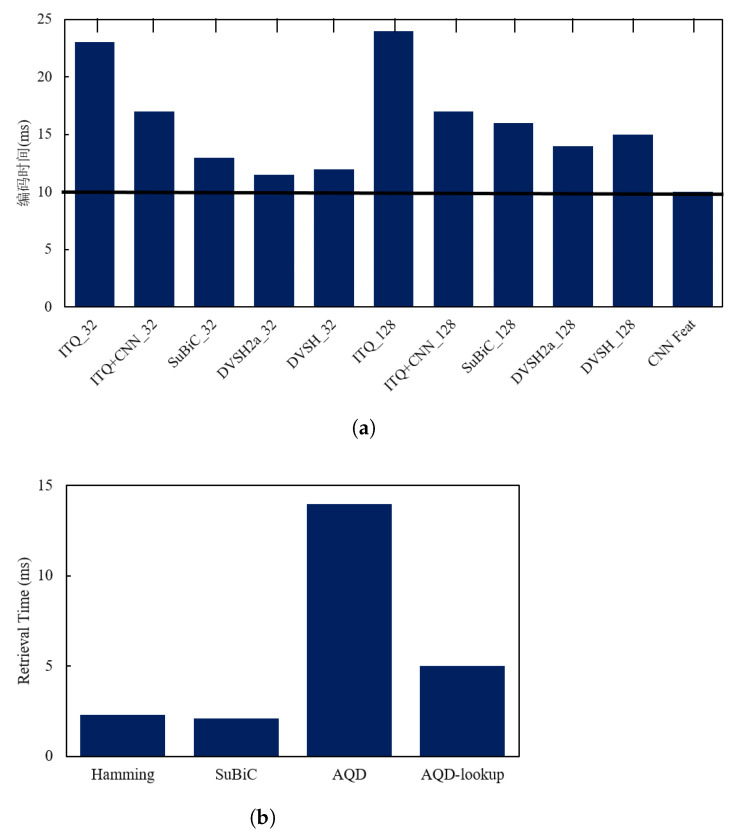
Time complexity analysis. (**a**) Comparison of encoding time of a single fabric image. (**b**) Comparison of retrieval time of a single fabric image.

An efficient retrieval system not only needs to output accurate retrieval results, but also must respond quickly to retrieval needs; that is, it must also have high timeliness. To demonstrate the efficiency of DVSH, we first compare the single image encoding times of different methods when the encoding length is 32 or 128 bits, including iterative quantization (ITQ) [27], ITQ+CNN [27], SuBiC [28] and DVSH2a (mentioned in the section of ablation study). Among them, ITQ and ITQ+CNN are two quantization-based dimensionality reduction methods, SuBic is a structured hashing method and DVSH2a is a variant of DVSH. The comparison results of the encoding time of the above methods are shown in Figure 8a, where “CNN Feat” represents the time when the convolutional neural network (Base Network) extracts features. As can be seen in the figure, the hash-based methods, SuBic and DVSH, consumed more time than CNN Feat because the encoding process takes a certain amount of time. Since more nodes will take more quantization time, the DVSH_128 encoding process takes more time than DVSH_32. However, the time consumption of high-dimensional feature extraction accounted for more than 70% of the total time-consuming, and the DVSH encoding time only accounted for less than 30%. DVSH is less than 10% more time-consuming than DVSH2a, indicating that the structural network module and the variational network module do not add too much computation.

Similarity measurement is another time-consuming link in CBIR. The hash method maps the similarity measure to the Hamming space, and then uses a simple and efficient XOR operation to calculate the similarity between binary codes. Quantization-based methods such as DVSQ [29] employ AQD [30] to compute the inner product between features to measure their similarity. SuBiC [28] computes asymmetric distances between features directly using the network output, thereby quickly computing similarities. In the experiment, the parameters of SuBiC and AQD were configured according to reference [28] and reference [29], respectively. The experimental results are shown in Figure 8b, which show that the query speed of AQD was seven times that of Hamming and SuBiC, and the time consumption of Hamming and and that of SuBiC were not very different. In retrieval, SuBiC only needs to perform a certain number of addition operations and use real-valued query codes. Although the time consumption is slightly lower than that of the Hamming, its calculation process takes up a lot of memory. AQD-lookup adopts the AQD of the code table, which uses the pre-calculated M × K code table, and directly uses the code table to query the distance between the two subspaces, which greatly shortens the time for quantizing the product. However, AQD-lookup needs to allocate memory on the code table of each query, increasing memory consumption, and the retrieval speed is lower than Hamming. In conclusion, the overall retrieval efficiency of DVSH is more suitable for efficient fabric image retrieval.

### 5.5. Comparisons

This section verifies the rationality and superiority of the proposed hashing model through horizontal comparison experiments. Some SOTA (state-of-the-art) deep hashing methods are compared, including: three hashing methods based on triplet loss supervised learning (DRSCH [31], DSRH [32], DNNH [33]), four supervised methods based on pairwise similarity loss (CNNH+ [34], DSH [35], DHN [11], IDHN [36]) and seven methods based on custom similarity guidance (CSQ [37], ISDH [38], DSHSD [39], SuBiC [28], MVL [40], UDHNR [41] and VTDH [42]. SuBiC is similar to DVSH, but it uses a sigmoid and threshold to binarize the output features to generate hash codes.

**Figure 9:**
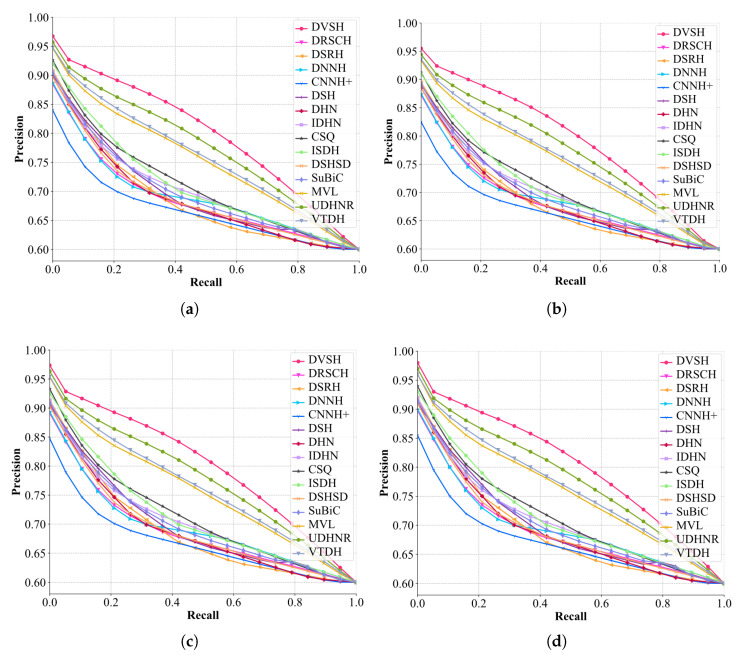
PR curves of different methods for fabric image retrieval (different code lengths): (**a**) 32 bits; (**b**) 64 bits; (**c**) 128 bits; (**d**) 256 bits.

**Figure 10:**
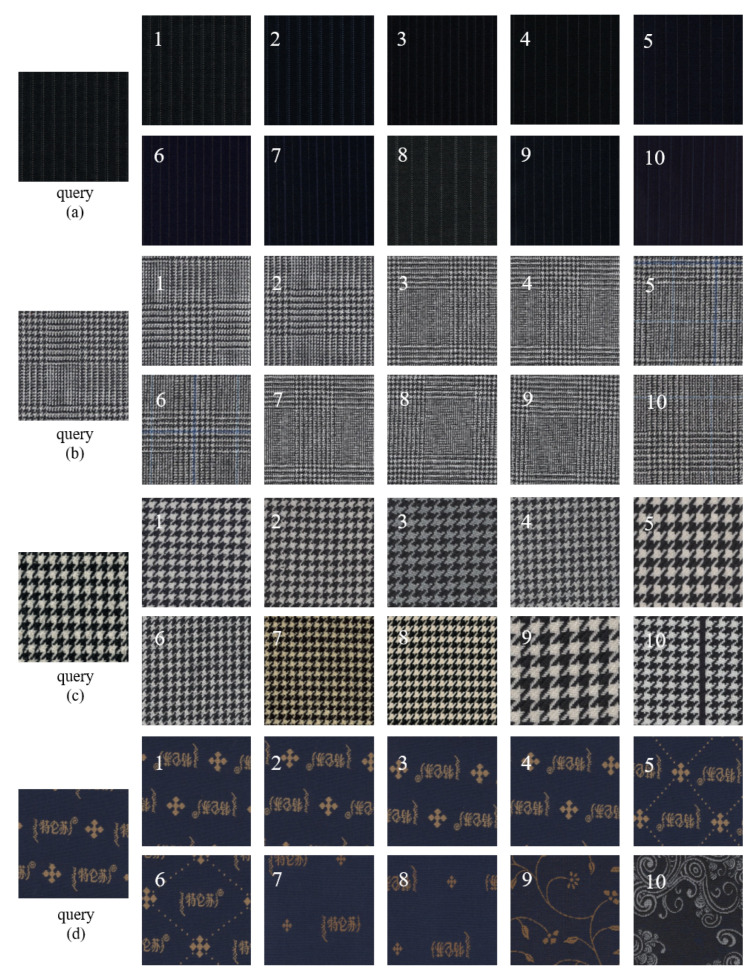
Several examples of fabric image retrieval. (**a**) Inlaid stripes; (**b**) Dobby; (**c**) Houndstooth; (**d**) Printed fabric (The pattern on the fabric appears to be “printed”).

The comparison results of mAP and $NDCG_{50}$ of different hashing methods are shown in Table 4. It can be observed from the results that methods based on triplet loss are generally more stable than methods based on pairwise similarity loss, proving that methods based on triplet loss learn more similarity information. However, the DVSH proposed in this paper not only utilizes the discriminative classification of fabrics to guide hash learning, but also uses list-wise learning to embed more similarity information in the generated hash codes. List-wise learning is guided by the similarity of a batch of fabric images, and its learning objective is more stringent than pairwise and triplet losses. The variational network module and the structural network module are used in the DVSH model to reduce the learning difficulty, and the content of the model’s learning is closer to the ideal fabric similarity arrangement. DVSH far outperformed other methods in $NDCG_{50}$, indicating that the hash code generated by DVSH contained more fabric fine-grain similarity information, and thus achieved a higher ranking score. As shown in Figure 9, the trends of PR curves for different code length models show consistency, illustrating the robust performance of the contrasting methods for fabric retrieval. The area under the PR curve corresponding to DVSH is higher than those of other methods at all encoding lengths, which verifies the superiority of the proposed method. In addition, all experimental results show that the longer the code length, the more fabric similarity information can be inherited, and thus the better the retrieval performance that can be obtained. It is worth noting that methods using soft similarity, such as IDHN and CSQ, achieved better retrieval results than other methods, indicating that more fine-grained similarity measurement methods can improve the retrieval performance of fabric images. Compared with the soft similarity, a more fine-grained measurement method was adopted, and more superior performance was obtained. The experimental results once again proved the validity of the fabric fine-grained similarity defined in this paper. Some retrieval samples are shown in Figure 10. The precise retrieval results show that DVSH has excellent performance for fabric image retrieval.

## 6. Conclusions

In this paper, a novel method for fabric image retrieval based on variational and structural hashing was proposed. To narrow the gap between fabric images and similarities, we designed a CNN with a compact structure and cross-shortcut connections, which is regarded as the base network of the hashing model. To overcome the problems of probabilistic missing and difficult training in classical hashing, we introduced a variational network module and structural network module into the hashing model (named DVSH). Then, a fine-grained similarity was defined to measure the similarity between two fabric images. To incorporate the defined fine-grained similarity into hash learning, we employed list-wise learning to complete similarity embeddings in mini-batches. The results of ablation experiments showed that the absence of any module will cause the performance of the model to decline, which verified the necessity and effectiveness of each module in DVSH. Through time complexity analysis, the single image encoding time of DVSH was only 16 milliseconds, which verified the real-time performance of the method. The retrieval performances of different hashing methods were compared, and DVSH achieved the best performance in different coding lengths, which verified the superiority of the method for fabric image retrieval. The method proposed in this paper has been successfully applied in cooperative enterprises.

## Figures and Tables

**Table 1:** Textile color classification standard.

	Black	Grey	White	Red		Orange	Yellow	Green	Cyan	Blue	Purple
h_min	0	0	0	0	156	11	26	35	78	100	125
h_max	180	180	180	10	180	25	34	77	99	124	155
s_min	0	0	0	43		43	43	43	43	43	43
s_max	255	43	30	255		255	255	255	255	255	255
v_min	0	46	221	46		46	46	46	46	46	46
v_max	46	220	255	255		255	255	255	255	255	255

**Table 2:** The retrieval performance of DVSH under different parameter configurations.

	$\lambda_{1}$	$\lambda_{2}$	mAP				${\mathrm{NDCG}}_{50}$			
			32 Bits	64 Bits	128 Bits	256 Bits	32 Bits	64 Bits	128 Bits	256 Bits
fix $\lambda_{2}$	$10^{-6}$	$10^{-3}$	0.662	0.672	0.698	0.711	0.609	0.612	0.628	0.640
	$10^{-5}$	$10^{-3}$	0.704	0.745	0.761	0.773	0.648	0.693	0.708	0.696
	$10^{-4}$	$10^{-3}$	0.741	0.795	0.815	0.824	0.667	0.716	0.742	0.742
	$10^{-3}$	$10^{-3}$	**0.825**	**0.853**	**0.876**	**0.879**	**0.743**	**0.785**	**0.806**	**0.800**
	$10^{-2}$	$10^{-3}$	0.753	0.807	0.827	0.835	0.693	0.751	0.744	0.752
	$10^{-1}$	$10^{-3}$	0.715	0.763	0.784	0.793	0.658	0.694	0.721	0.737
	1	$10^{-3}$	0.682	0.711	0.736	0.742	0.634	0.640	0.662	0.683
fix $\lambda_{1}$	$10^{-3}$	1	0.479	0.524	0.615	0.649	0.426	0.466	0.560	0.591
	$10^{-3}$	$10^{-1}$	0.671	0.772	0.824	0.829	0.590	0.679	0.725	0.738
	$10^{-3}$	$10^{-2}$	0.783	0.834	0.861	0.868	0.689	0.759	0.766	0.773
	$10^{-3}$	$10^{-4}$	0.819	0.845	0.874	0.878	0.721	0.761	0.795	0.799
	$10^{-3}$	$10^{-5}$	0.821	0.842	0.875	0.876	0.739	0.758	0.788	0.788
	$10^{-3}$	$10^{-6}$	0.781	0.810	0.824	0.849	0.687	0.729	0.742	0.764

The optimal value of each column of experimental results in the table is marked with black, and the worst value is marked with underline.

**Table 3:** Performance comparison of DVSH and its variants.

Name	mAP				${\mathrm{NDCG}}_{50}$			
	32 Bits	64 Bits	128 Bits	256 Bits	32 Bits	64 Bits	128 Bits	256 Bits
DVSH1a	0.803	0.825	0.847	0.852	0.723	0.743	0.771	0.767
DVSH1b	0.814	0.832	0.854	0.863	0.733	0.757	0.786	0.777
DVSH2a	0.792	0.799	0.811	0.824	0.713	0.735	0.730	0.750
DVSH	**0.825**	**0.853**	**0.876**	**0.879**	**0.743**	**0.785**	**0.806**	**0.800**

The optimal value of each column of the experimental results in the table is marked with black, and the worst value is marked with an underline.

**Table 4:** Comparative results of different deep hashing methods for fabric image retrieval.

Methods	mAP				NDCG50			
	32 Bits	64 Bits	128 Bits	256 Bits	32 Bits	64 Bits	128 Bits	256 Bits
DRSCH [31]	0.697	0.719	0.738	0.744	0.599	0.611	0.649	0.647
DSRH [32]	0.689	0.725	0.739	0.742	0.593	0.624	0.628	0.653
DNNH [33]	0.692	0.729	0.744	0.754	0.602	0.620	0.640	0.648
CNNH+ [34]	0.661	0.675	0.691	0.708	0.575	0.581	0.601	0.616
DSH [35]	0.691	0.722	0.748	0.761	0.608	0.636	0.636	0.670
DHN [11]	0.708	0.735	0.745	0.755	0.623	0.647	0.656	0.664
IDHN [36]	0.736	0.756	0.784	0.797	0.633	0.650	0.682	0.693
CSQ [37]	0.742	0.767	0.782	0.803	0.653	0.667	0.688	0.707
ISDH [38]	0.735	0.755	0.775	0.789	0.647	0.642	0.659	0.671
DSHSD [39]	0.708	0.726	0.741	0.755	0.602	0.617	0.652	0.642
SuBiC [28]	0.702	0.735	0.764	0.774	0.597	0.647	0.665	0.673
MVL [40]	0.751	0.771	0.795	0.809	0.687	0.712	0.739	0.741
UDHNR [41]	0.768	0.786	0.814	0.822	0.692	0.717	0.732	0.735
VTDH [42]	0.759	0.785	0.801	0.814	0.684	0.715	0.727	0.733
DVSH	**0.825**	**0.853**	**0.876**	**0.879**	**0.743**	**0.785**	**0.806**	**0.800**

The optimal value of each column of experimental results in the table is marked with black, and the worst value is marked with an underline.

## Data Availability

Not applicable.
